# Serum elabela and apelin levels during different stages of chronic kidney disease

**DOI:** 10.1080/0886022X.2020.1792926

**Published:** 2020-07-25

**Authors:** Xuehong Lu, Shengmao Liu, Rumei Luan, Wenpeng Cui, Yu Chen, Yixian Zhang, Yue Lu, Hong Zhang, Lin Shi, Lining Miao, Feng Xu

**Affiliations:** a Department of Nephrology, The Second Hospital of Jilin University, Changchun, China; b Department of Endocrinology, Huaian First People’s Hospital, Nanjing Medical University, Huai’an China; c Department of Pediatrics, Shandong Provincial Hospital affiliated to Shandong University, Jinan, China

**Keywords:** Elabela (ELA), Apelin, chronic kidney disease (CKD), estimated glomerular filtration rate (eGFR)

## Abstract

**Purpose:**

The association of serum elabela (ELA) and apelin with the progression of chronic kidney disease (CKD) is unknown. We determined if serum ELA and apelin levels were associated with CKD stage.

**Methods:**

This observational study involved 60 CKD patients and 20 healthy, age-, race-, and gender-matched controls. The participants were grouped according to renal function as follows: normal control group, CKD1 group (stage-1 CKD, 20 patients), CKD3 group (stage-3 CKD, 20 patients), and CKD5 group (stage-5 CKD, 20 patients) in accordance with the Kidney Disease Outcomes – Quality Initiative criteria. We recorded the demographic, clinical, and biochemical data of all participants. Serum ELA and apelin levels were measured using commercially available enzyme-linked immunosorbent assays.

**Results:**

Serum ELA levels gradually and significantly declined with decreases in the estimated glomerular filtration rate (eGFR). Serum ELA showed significant negative correlations with serum creatinine (*r* = −0.529, *p* < .001), blood urea nitrogen (*r* = −0.575, *p* < .001), systolic blood pressure (*r* = −0.455, *p* < .001), and diastolic blood pressure (*r* = −0.450, *p* < .001), and significant positive correlations with hemoglobin (*r* = 0.523, *p* < .001) and eGFR (*r* = 0.728, *p* < .001). Multiple regression analysis showed that eGFR independently influenced serum ELA levels. No significant association was found between serum apelin levels and CKD progression.

**Conclusion:**

In CKD patients, serum ELA levels decreased with decreasing eGFR. This finding may provide a new target for the prediction, diagnosis, and staging of CKD.

## Introduction

The overall prevalence of chronic kidney disease (CKD) in the world is estimated to be 13.4%, while that of advanced CKD (stages 3–5) is estimated to be 10.6% [1]. All stages of CKD are associated with increased cardiovascular morbidity, premature mortality, poor quality of life, and substantial health-care resource utilization. Early-stage CKD is usually asymptomatic. Once CKD progresses to stage 3 however, the risks of complications and deterioration to end-stage renal disease (ESRD) are significantly increased. Multisystem dysfunction and complications develop, and renal replacement therapy is necessary in stage-5 CKD. Therefore, novel biological markers for the progression of CKD are required to not only improve the accuracy of risk prediction but also discover new pathways of CKD progression [2,3].

Apelin is an endogenous ligand of APJ receptors, which are G protein-coupled receptors related to angiotensin II receptors (type AT1a) [4]. Studies have shown that the apelin/APJ system is widely distributed in the body and are involved in important physiological functions, including cardiovascular, renal, energy metabolism, and fluid homeostasis [5–10]. Serum apelin levels have been reported to be closely related to progression of a variety of kidney diseases [11–14]. Elabela (ELA), also called Apela or Toddler, is a highly conserved, 54-amino acid-long polypeptide that was identified in human embryonic stem cells in 2013 [15,16]. ELA has been demonstrated to be an earlier ligand for APJ receptors than apelin [17,18]. Studies in adult rats have shown that unlike the wide tissue distributions of apelin and APJ, ELA is expressed exclusively in the kidneys [19,20]. A recent study showed that ELA reduces changes in the glomerular structure, inhibits fibrosis-related gene expression in the kidneys, and reduces renal fibrosis in high-salt diet-induced hypertensive rats [21].

We hypothesized that serum apelin and ELA levels may vary with the severity of CKD and may serve as biomarkers for CKD progression. The present study, therefore, aimed to measure the serum apelin and ELA levels in patients with different stages of CKD and determine whether these levels were associated with CKD stages and clinical characteristics.

## Materials and methods

### Study subjects and ethics statement

This observational study enrolled patients who were evaluated for CKD stages 1, 3, and 5 at the time of their first visit to the Second Hospital of Jilin University between January 2017 and December 2018. The inclusion criteria were as follows: (1) CKD diagnosed according to the Kidney Disease Outcomes – Quality Initiative (KDOQI) criteria [22]; (2) CKD categorized as stage 1, 3, or 5 according to the KDOQI staging system [22]; and (3) age ≥ 18 years. Patients were excluded from the study if they (1) were receiving ongoing treatment with hemodialysis or peritoneal dialysis, (2) had undergone renal transplantation, or (3) had liver disease, heart failure, stroke, rheumatic disease, autoimmune disease, or malignant tumors, were pregnant, or were taking steroids. In addition to these subjects, 20 age- and gender-matched volunteers with no known kidney disease and a negative result on urinary dipstick analysis were enrolled to serve as healthy controls.

The study was approved by the ethics committee of the Second Hospital of Jilin University (approval number: 2019041), and complied with the tenets of the Declaration of Helsinki. The study protocol was explained to all patients, and each participant provided written informed consent.

### Study groups

The estimated glomerular filtration (eGFR) was calculated using the CKD Epidemiology Collaboration (CKD-EPI) equation. Patients and volunteers were divided into four groups as follows: normal control group (*n* = 20), CKD1 group (CKD stage 1, eGFR ≥ 90 mL/min/1.73 m^2^, *n* = 20), CKD3 group (CKD stage 3, eGFR = 30–59 mL/min/1.73 m^2^, *n* = 20), and CKD5 group (CKD stage 5, eGFR < 15 mL/min/1.73 m^2^, *n* = 20). The proportion of secondary renal failure was similar in each study group (Table S1 in the Supplementary Appendix).

### Data collection

Demographic, clinical, and biochemical data of the subjects were obtained from medical records and queries (e.g., gender, age, and history of hypertension), physical examination (e.g., height, weight, and blood pressure), and laboratory tests (e.g., hemoglobin (Hb), serum albumin, blood urea nitrogen (BUN), serum creatinine (Cre), ELA and apelin levels). Body mass index (BMI) was defined as weight/height^2^ (kg/m^2^). Blood pressure was measured in the right arm, with the subjects in a sitting position after a 10-min rest period; a mercury sphygmomanometer and a stethoscope were used for the measurement, following the recommendations of the American Heart Association [23]. Systolic and diastolic blood pressures were defined at the first and fifth phases of Korotkoff sounds, respectively. Blood samples were collected before taking any antihypertensive medications. The serum levels of Hb, BUN, and Cre were analyzed using UniCel DxH 800 (Beckman Coulter, Miami, FL) and Hitachi 008AS Autoanalyzer (Hitachi, Tokyo, Japan). Enzyme-linked immunosorbent assay was used to evaluate serum ELA and apelin levels. Venous blood samples were collected in EDTA/acetic acid-containing tubes and centrifuged at 3000 rpm for 10 min at 4C. ELA and apelin assays were performed using the ELABELA (human)-EIA kit (Peninsula Laboratories International Inc., San Carlos, CA, USA) and Apelin (human) C-Terminus EIA kit (RayBiotech Inc., Norcross, GA, USA) according to the manufacturers’ instructions.

### Statistical analysis

All data were analyzed using SPSS software, version 20.0 (SPSS Inc., Chicago, IL, USA). Depending on the data distribution and homogeneity of variances, differences between groups were analyzed using one-way analysis of variance, the chi-square test, or the Kruskal-Wallis test. Multiple linear regression, Pearson correlation, and Rank correlation analyses were used to identify significant correlations among the variables. *p* values of <.05 were considered to indicate statistically significant differences.

## Results

### Subject characteristics

The characteristics of the study participants have been presented in Table 1. Age, gender, and body-mass index did not significantly differ among the various study groups. Compared with the normal control group, the three CKD groups had significantly higher systolic blood pressure (SBP) and diastolic blood pressure (DBP) and clearly lower albumin and hemoglobin levels. SBP was highest in the CKD5 group.

**Table 1.** Demographic, clinical, and biochemical characteristics of all study subjects.

	Normal control	CKD1	CKD3	CKD5	*p* value
Age (years)	51.95 ± 10.44	48.20 ± 8.49	50.85 ± 12.48	52.40 ± 16.72	.576
Sex (male/female)	10/10	11/9	10/10	10/10	.985
BMI (kg/m^2^)	23.10 ± 1.61	23.06 ± 3.78	25.71 ± 3.61	25.54 ± 3.39	.054
SBP (mm Hg)	118.60 ± 4.49	135.60 ± 13.7^a^	149.05 ± 16.37^a^	165.80 ± 14.22^a^^b^	<.001
DBP (mmHg)	80.20 ± 4.02	89.75 ± 7.08	99.85 ± 13.00^a^	106.30 ± 12.13^a^^b^	<.001
Hb (g/L)	144.70 ± 14.09	136.00 ± 22.57	122.55 ± 12.08^a^	92.50 ± 18.57^a^^bc^	<.001
Albumin (g/L)	43.41 ± 2.96	37.67 ± 8.09	32.05 ± 10.37^a^	34.49 ± 6.53^a^	<.001
BUN (mmol/L)	5.59 ± 1.48	4.89 ± 1.35	10.34 ± 5.19^a^^b^	23.49 ± 9.17^a^^bc^	<.001
Cre (μmol/L)	59.85 ± 10.84	64.80 ± 14.39	135.25 ± 63.25^a^^b^	724.80 ± 309.73^a^^bc^	<.001
eGFR (mL/min/1.73 m^2^)	112.14 ± 18.05	110.01 ± 19.19	44.50 ± 9.92^a^^b^	7.58 ± 2.83^a^^bc^	<.001
Elabela (ng/mL)	6.14 ± 1.03	5.39 ± 1.91	3.81 ± 1.12^a^^b^	2.67 ± 1.01^a^^bc^	<.001
Apelin (ng/mL)	3801.10 ± 902.21	3520.75 ± 1002.97	3615.90 ± 1012.54	3547.05 ± 1192.16	.825

CKD: chronic kidney disease; CKD1, CKD3, and CKD5, CKD stages one, three, and five; BMI: body-mass index; SBP: systolic blood pressure; DBP: diastolic blood pressure; BUN: blood urine nitrogen; Cre: serum creatinine; eGFR: estimated glomerular filtration rate. Data are expressed as mean ± SD.

^a^ *p* < .05 vs. the normal control group. ^b^*p* < .05 vs. the CKD1 group. ^c^*p* < .05 vs. the CKD3 group.

### Serum ELA and apelin levels

**Figure 1.**
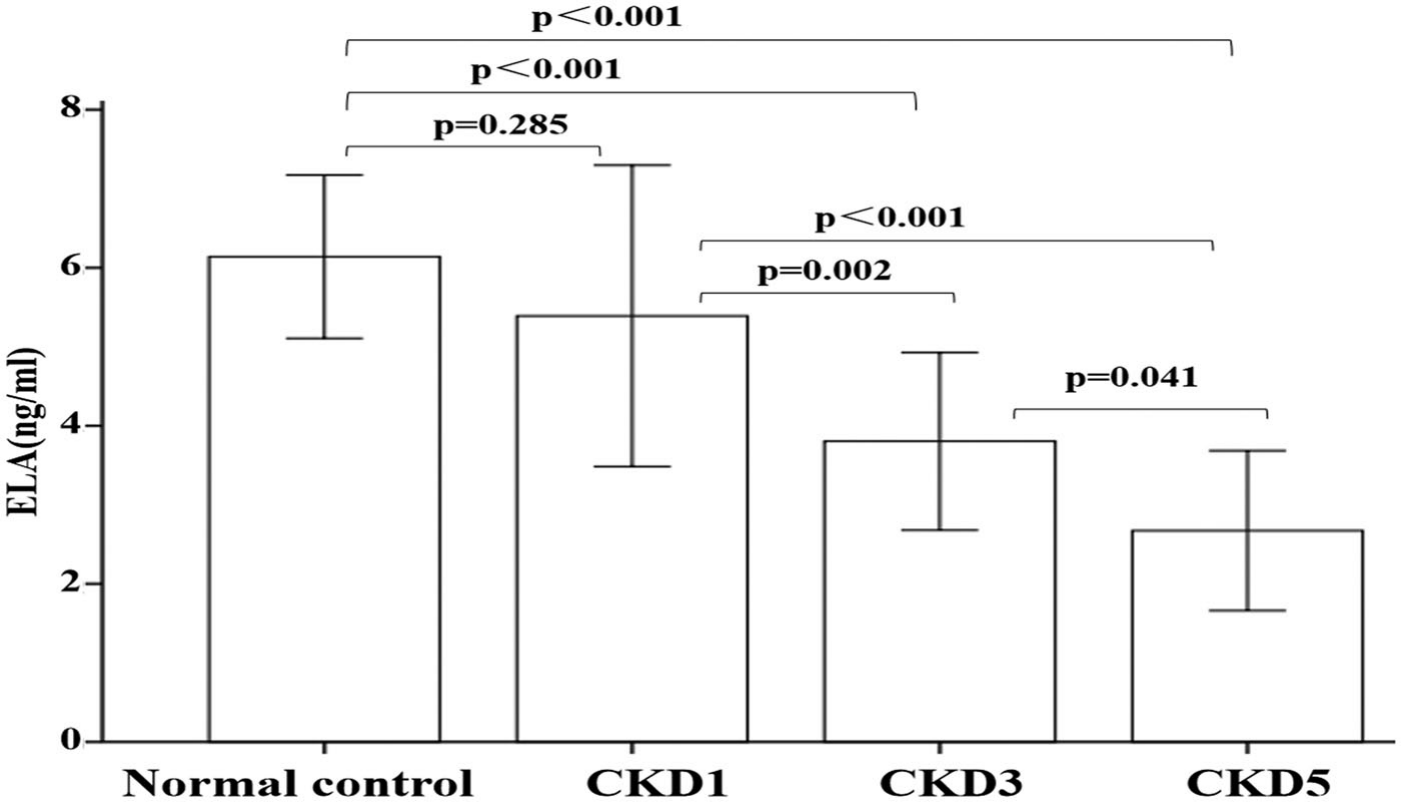
Serum ELA levels in each study group. Data are expressed as mean ± SD.

Serum ELA levels gradually declined with decreasing eGFR (Figure 1). Pairwise comparisons showed that serum ELA levels were significantly higher in the normal control and CKD1 groups than in the CKD3 and CKD5 groups (*p* < .05 for all) and were higher in the CKD3 group than in the CKD5 group (*p* = .041). The other comparisons had *p*-values larger than .05. In contrast, serum apelin levels did not differ between study groups.

### Association of ELA with CKD progression

We next studied the relationship of serum ELA levels with other variables (Table 2). Simple correlation analyses (*P*-values between variables) showed that serum ELA had a significant negative correlation with SBP (*r* = −0.455, *p* < .001), DBP (*r* = −0.450, *p* < .001), BUN (*r* = −0.575, *p* < .001), and serum creatinine (*r* = −0.529, *p* < .001) and a significant positive correlation with hemoglobin (*r* = 0.523, *p* < .001) and eGFR (*r* = 0.728, *p* < .001). To control for potential confounders, we performed stepwise multiple linear regression analysis with ELA as the dependent variable and all of the other aforementioned variables as independent variables. The results showed that eGFR was an independent predictor of ELA (Table 3).

**Table 2.** R-values and P-values of correlations among the study variables.

	ELA	Age	Sex	SBP	DBP	Hb	Alb	BUN	Cre	eGFR
Age	–0.045									
	0.689									
Sex	–0.037	0.105								
	0.742	0.356								
SBP	–0.455	0.031	–0.032							
	<0.001	0.782	0.778							
DBP	–0.450	–0.036	–0.103	0.802						
	<0.001	0.749	0.363	<0.001						
Hb	0.523	0.039	–0.250	–0.559	–0.519					
	<0.001	0.732	0.025	<0.001	<0.001					
Alb	0.186	–0.067	–0.025	–0.350	–0.327	0.418				
	0.099	0.555	0.823	0.001	0.003	<0.001				
BUN	–0.575	0.200	–0.136	0.543	0.527	–0.619	–0.125			
	<0.001	0.076	0.229	<0.001	<0.001	<0.001	0.268			
Cre	–0.529	–0.028	–0.190	0.522	0.479	–0.690	–0.116	0.826		
	<0.001	0.808	0.091	<0.001	<0.001	<0.001	0.306	<0.001		
eGFR	0.728	–0.078	0.034	–0.722	–0.655	0.680	0.387	–0.751	–0.735	
	<0.001	0.489	0.767	<0.001	<0.001	<0.001	<0.001	<0.001	<0.001	
Apelin	0.129	–0.048	0.018	–0.011	–0.022	0.055	–0.005	–0.143	–0.065	0.063
	0.255	0.675	0.871	0.923	0.843	0.629	0.966	0.205	0.564	0.579

ELA: elabela; SBP: systolic blood pressure; DBP: diastolic blood pressure; Hb: hemoglobin; Alb: albumin; BUN: blood urine nitrogen; Cre: serum creatinine; eGFR: estimated glomerular filtration rate. The first row for each variable represents the *r* value, and the second row represents the *p* value.

**Table 3.** Stepwise multiple linear regression analysis of ELA.

Variable	Partial regression coefficient	SE	Standard partial regression coefficient	t	*p*	95% CI of partial regression coefficient
Constant	2.501	0.258		9.696	<.001	(1.988, 3.015)
eGFR	0.029	0.003	0.728	9.376	<.001	(0.023, 0.035)

ELA: elabela; SE: standard error; CI: confidence interval; eGFR: estimated glomerular filtration rate. Multiple R-squared: 0.530; adjusted R-squared: 0.524.

## Discussion

In this study, we have revealed, for the first time, that serum ELA levels were significantly associated with the progression of CKD. Moreover, multiple regression analysis showed that serum ELA levels were independently associated with eGFR in our cohort of 60 CKD patients. In contrast, serum apelin levels were not significantly related with the progression of CKD.

Many studies have shown the importance of the apelin/APJ system in kidney diseases [24]. Serum apelin levels have been reported to be reduced in patients with autosomal dominant polycystic kidney disease [11] and increased in patients with stage-5 CKD, even after treatment with blood and peritoneal dialysis [12]. Additionally, apelin levels have been found to be elevated in patients with type 2 diabetes and in the kidneys of diabetic mice [13]. Conversely, other researchers have reported that renal apelin-13 levels are decreased in type 1 diabetic mice and increased after treatment with apelin-13 [14]. Although serum apelin levels were associated with CKD in the above studies, no statistically significant association between apelin levels and CKD progression was found in the present study. This difference may be related to interspecies differences, as the above studies were conducted on mice, while we assessed patients in comparison to healthy controls.

Recently, ELA, a novel endogenous ligand of APJ receptors, was discovered. Certain biological effects of ELA may be similar to those of apelin, such as lowering blood pressure [25] and promoting diuresis by activating APJ downstream signaling pathways [26]. However, ELA binds to APJ receptors prior to apelin [15,16,27] and is more effective than apelin in some respects. For instance, ELA promotes cell proliferation, reduces apoptosis [28], and stimulates angiogenesis [29]. Moreover, it can enhance glucose uptake and utilization, and improve insulin resistance [30].

The above results suggest that ELA may have protective roles in various kidney diseases. In rats with high-salt diet-induced hypertension, treatment with ELA–adeno-associated virus effectively reduced structural glomerular changes and prevented renal fibrosis by inhibiting the expression of fibrosis-related genes in the kidney [21]. In a renal ischemia-reperfusion (I/R) injury model, ELA-32 and ELA-11 significantly inhibited I/R injury-induced renal fibrosis, inflammation, apoptosis, and DNA damage response, and remarkably protected against the development of renal tubular lesions and renal dysfunction [31]. These data collectively imply that ELA plays a protective role in kidney diseases, specifically CKD. However, no study has as yet researched the relationship between serum ELA levels and CKD stages.

In the present study, we found that serum ELA levels were slightly, but not significantly, lower in patients with stage-1 CKD than in the normal controls. The serum ELA levels were still lower in patients with stage-3 CKD and were lowest in patients with stage-5 CKD. The ELA values ​​we observed in the CKD5 group are consistent with those reported by Masaki et al. [32] in ESRD patients on maintenance hemodialysis. This suggests that the abnormal ELA levels observed during maintenance dialysis may merely reflect the pre-dialysis levels. We noticed that serum ELA levels progressively decreased with decreasing eGFR, which suggests that a reduction in ELA levels could predict CKD progression. In addition, serum ELA levels were negatively correlated with SBP, DBP, BUN, and serum creatinine, and positively correlated with hemoglobin and eGFR. Recently, it was reported that ELA can lower the blood pressure [25,30]. Research has shown that ELA exerts a vasodilatory effect [33] by attenuating the potentiation of angiotensin II-induced vasopressor in mice [29,34]. Furthermore, stepwise multiple linear regression analysis demonstrated that eGFR was an independent predictor of the serum ELA level.

Interestingly, although ELA and apelin share the same ligand and similar signaling pathways, only the level of ELA, and not of apelin, was related to CKD progression. This difference may be attributable to the difference in the tissue distributions of ELA and apelin. Several studies in adult rats have shown that unlike the wide tissue distributions of apelin and APJ, ELA is expressed exclusively in the kidneys [19,20]. Therefore, considering the varying degrees of renal fibrosis in CKD patients, we speculated that the ELA level progressively declined with the aggravation of CKD. In contrast, the level of apelin, which is widely distributed in the body, was not related to the progression of CKD.

Considering the known functions of ELA in inhibiting renal fibrosis [21], promoting cell survival, and lowering blood pressure [15,16,35–37], we believe that it is reasonable to speculate that decreased serum ELA levels are not merely a marker of kidney damage but may if fact be a cause of the CKD progression. Therefore, more research is warranted to clarify the role of ELA in CKD. To our knowledge, this is the first study to evaluate serum ELA levels in patients with different stages of CKD before dialysis and demonstrate a significant correlation between ELA levels and CKD progression.

The present study does have certain limitations. First, the relatively small sample size may not represent the true relationship between serum ELA levels and CKD stages in all CKD patients. Second, experimental and interventional studies are needed to explore the mechanisms *via* which ELA is associated with certain kidney diseases and to identify causal relationships. Hence, the present results must be viewed as preliminary.

In conclusion, our study shows for the first time that serum ELA levels gradually decline in CKD patients and are significantly associated with eGFR. This finding may provide a new target for the prediction, diagnosis, and staging of CKD. Further studies aiming to determine whether the modulation of the ELA/APJ system results in any clinical improvement in CKD subjects would be highly desirable.

## Supplementary Material

Supplemental Material
