# Supplementary material for: Serum elabela and apelin levels during different stages of chronic kidney disease
Source: Ren Fail. 2020 Jul 25;42(1):667–72. doi: 10.1080/0886022X.2020.1792926 (PMC7470108; doi:10.1080/0886022X.2020.1792926)
Supplement: Supplemental Material [file IRNF_A_1792926_SM5381.pdf]

Table S1 The distribution of primary disease in stages of chronic kidney disease

| | CKD1（n=20） | CKD3（n=20） | CKD5（n=20） | P value |
|---|---|---|---|---|
| CGN | 10（50%） | 10（50%） | 11（55%） | 0.935 |
| Hypertension | 3（15%） | 4（20%） | 3（15%） | 0.889 |
| Diabetes | 2（10%） | 3（15%） | 3（15%） | 0.860 |
| CPN | 2（10%） | 2（10%） | 1（5%） | 0.789 |
| SLE | 2（10%） | 1（5%） | 1（5%） | 0.776 |
| HSP | 1（5%） | 0（0%） | 1（5%） | 0.437 |

CKD,chronic-kidney-disease;CGN,chronic glomerulonephritis;CPN,chronic pyelonephritis;SLE, systemic lupus erythematosus;HSP,henoch-schonlein purpura
